# Empowering Patients: A Multicomponent Workshop Improves Self-Management and Quality of Life in Chronic Pain

**DOI:** 10.3390/medsci13040319

**Published:** 2025-12-15

**Authors:** María Victoria Ruiz-Romero, María Begoña Gómez-Hernández, Ana Porrúa-Del Saz, María Blanca Martínez-Monrobé, Natalia Gutiérrez-Fernández, Almudena Arroyo-Rodríguez, Rosa Anastasia Garrido-Alfaro, Néstor Canal-Diez, María Dolores Guerra-Martín, Consuelo Pereira-Delgado

**Affiliations:** 1 San Juan de Dios del Aljarafe Hospital, Bormujos, 41930 Seville, Spain; mariavictoria.ruiz@sjd.es (M.V.R.-R.); mbghernandez@euef.comillas.edu (M.B.G.-H.); ana.porrua.delsaz@gmail.com (A.P.-D.S.); mbmmonrobe@euef.comillas.edu (M.B.M.-M.); nataliagutfer@gmail.com (N.G.-F.); rosagarridoalfaro@gmail.com (R.A.G.-A.); cmpereira@euef.comillas.edu (C.P.-D.); 2 San Juan de Dios Foundation, 28015 Madrid, Spain; 3 Health Sciences Department, San Juan de Dios School, Comillas Pontifical University, Bormujos, 28036 Sevilla, Spain; 4 Semillero de Investigación José Bueno O.H., San Juan de Dios University Nursing Center, Bormujos, 41930 Seville, Spain; nesgercandie@gmail.com; 5 Department of Nursing, Faculty of Nursing, Physiotherapy and Podiatry, University of Seville, 41009 Seville, Spain; 6 Seville Institute of Biomedicine (IBiS), 41013 Seville, Spain

**Keywords:** quality of life, self-care, resilience, anxiety, health outcomes

## Abstract

Background: Chronic pain is a prevalent and disabling condition, affecting 20–30% of the global population, which requires multidisciplinary approaches integrating non-pharmacological therapies and promoting patient engagement in self-management. Objective: To describe the structure, content, outcomes, and lessons learned from multicomponent workshops for chronic non-cancer pain using non-pharmacological therapies. Methods: A quasi-experimental before–after study was conducted in patients attending a chronic pain workshop at San Juan de Dios Hospital (Bormujos, Seville, Spain) between November 2021 and May 2024, with a 3-month follow-up, Validated scales and an ad hoc patient survey were administered at baseline, immediately post-workshop, and at 3-month follow-up. Furthermore, comparative analysis was conducted 4 months before and after the intervention for emergency visits and consultations, medication consumption, and employment status. Analyses employed Chi-square or Fisher’s exact tests (categorical variables); student’s *t*-tests or Mann–Whitney U (between-group); paired *t*-tests or Wilcoxon (within-group pre–post); and effect sizes (Cohen’s d, Rosenthal’s r). Significance was set at *p* < 0.05. Results: 197 patients completed the workshop; 178 (90.4%) were women, mean age: 55.0; 114 (57.9%) had fibromyalgia. Reductions were observed in: pain (scale 0–10) (baseline: 7.0; end of workshop: 5.0; 3 months: 5.0; *p* < 0.001); anxiety (13.0; 9.0; 11.0; *p* < 0.001); and depression (11.4; 7.2; 6.8; *p* < 0.001) (scales 0–21). Increases were noted in: well-being (scale 0–10) (4.0; 6.0; 5.0; *p* < 0.001); quality of life (scale 0–1) (0.399; 0.581; 0.556; *p* < 0.001); health status (scale 0–100) (40.0; 60.0; 60.0; *p* < 0.001); self-esteem (scale 9–36) (23.5; 27.1; 26.6; *p* < 0.001); and resilience (scale 6–30) (17.0; 18.0; 18.0; *p* = 0.002, *p* < 0.001). PROMs were completed by 189 patients at the end of the workshop and 110 at 3 months: pain decreased (end of workshop: 76.7%; 3 months: 80.7%); medication decreased (80.5%; 78.1%); and habits improved (87.2%; 87.6%). 40 patients (37.4%) reduced emergency visits and scheduled consultations. Overall satisfaction: 9.7. Conclusions: The workshop enhanced patients’ self-management and produced improvements in pain, quality of life, emotional well-being, and self-esteem, with effects maintained at 3 months.

## 1. Introduction

Chronic pain (CP) is a prevalent and disabling condition, affecting 20–30% of the global population [1,2,3], which requires multidisciplinary approaches integrating non-pharmacological therapies and promoting patient engagement in self-management.

According to data from the Chronic Pain Barometer conducted in Spain in 2022 [3], 25.9% of the Spanish population suffers from CP, which accounts for over 9 million people. The prevalence is higher in women (30.5% vs. 21.3% in men), and the average age is 51.5 years.

CP is predominantly driven by highly prevalent musculoskeletal disorders, particularly low back pain, neck pain, and osteoarthritis, which consistently rank among the leading global causes of years lived with disability, as demonstrated by the Global Burden of Disease studies [4]. These conditions frequently coexist with central sensitization and comorbid anxiety or depression, which exacerbate pain perception, functional impairment, and the transition to chronicity.

Large epidemiological surveys in Europe and Spain likewise show a higher prevalence and impact of CP among women, older adults, individuals with lower educational attainment or income, those outside the labor market, and persons with mental health disorders, reflecting the interaction of biological mechanisms (e.g., sex-related differences in pain processing, hormonal influences), psychosocial vulnerability (catastrophizing, stress, mood disturbances), and social determinants (physically demanding work, caregiving responsibilities, socioeconomic disadvantage) [1,3,5].

The higher burden observed in women has been consistently documented and may reflect sex-related differences in pain modulation, increased prevalence of anxiety and depression, and greater exposure to caregiving and stress-related roles, as evidenced by large population-based studies [5,6]. Patients with CP have a high consumption of healthcare resources. Spaniards over 14 years old with CP who take pain medication increased from 54% in 2014 to 67% in 2020. Consumption is higher in women, especially older women, and in individuals with lower socioeconomic and educational levels [5].

Of the patients surveyed in the Chronic Pain Barometer, 42.1% visited the healthcare system in the last month, mainly primary care (86.7%), traumatology (69.4%), and emergency services (45%). 28.6% took 4–5 months of sick leave in the last year; 32.2% quit their job [3].

Management strategies based solely on pharmacological therapies have limited effectiveness, as they do not always control pain and can produce side effects, including addiction in the case of opioids, which raises questions about the risk-benefit ratio of treatments [7,8]. Currently, the trend is to combine pharmacological therapies with Non-Pharmacological Therapies (NPTs) in a comprehensive approach to treat CP [9,10,11,12,13,14,15,16,17].

NPTs comprise evidence-based psychological, educational, behavioral, and lifestyle interventions, such as cognitive behavioral therapy, acceptance and commitment therapy, mindfulness-based strategies, relaxation techniques, therapeutic exercise, and pain neuroscience education, designed to modulate the cognitive, emotional, and physiological mechanisms sustaining CP. These interventions are frequently delivered through multicomponent programs that integrate several therapeutic modalities within a single protocol, typically encompassing psychological, educational, behavioral, mind–body, and lifestyle components [12,18,19].

Furthermore, many programs also adopt a multidisciplinary framework, involving coordinated participation of clinicians from medicine, psychology, nursing, and physiotherapy [4,7,8]. Collectively, these approaches aim not only to reduce pain but also to enhance quality of life (QoL) and promote active patient involvement in self-management [12,20].

Recent systematic evidence demonstrates that such programs produce improvements in pain intensity, catastrophizing, anxiety, depression, physical function, and QoL [18].

The theoretical foundation of these interventions is rooted in the biopsychosocial model, which conceptualizes CP as emerging from interactions among nociceptive processes, maladaptive cognitions (e.g., catastrophizing), emotional distress (e.g., anxiety and depression), and contextual or lifestyle factors. Evidence from multicomponent programs (including cognitive behavioral therapy, acceptance and commitment therapy, and mindfulness-based interventions) demonstrates that targeting these domains enhances self-efficacy, emotion regulation, and adaptive coping, thereby contributing to clinically meaningful improvements across multiple outcomes. [18,21,22,23].

Evidence indicates that multicomponent workshops are effective in reducing pain [12,21,22,23], decreasing catastrophizing [23], reducing analgesic consumption [12,14,22]; improving anxiety and depression [12,14,23]; improving sleep quality [21]; and increasing QoL [12,14,17,21].

These outcomes are clinically relevant, as psychological distress, low self-esteem, impaired sleep, and reduced resilience are well-established predictors of pain severity, disability, and healthcare utilization in CP populations. Improvements in these domains therefore represent meaningful functional changes and are consistent with the multidimensional nature of CP and current clinical recommendations [18].

Our hospital, which serves 300,000 inhabitants from 28 municipalities, has been conducting programs (workshops) for the control of non-oncological CP with NPTs since 2016, having completed 25 editions with 390 patients. The objective was to describe what these workshops consist of (structure and content), health outcomes, and the main lessons learned.

The specific objectives of this study were: (1) to evaluate whether participation in the multicomponent workshop produced improvements in pain, well-being, QoL, self-esteem, resilience, anxiety, depression, medication use, emergency department visits, and scheduled outpatient consultations relative to baseline, and (2) to determine whether these effects were maintained at the 3-month follow-up.

## 2. Materials and Methods

### 2.1. Design

A quasi-experimental, single-group pretest–posttest design with a 3-month follow-up was used to evaluate the effectiveness of a multicomponent psychoeducational workshop for patients with chronic non-oncological pain. This within-subjects design enabled the assessment of changes in pain intensity, emotional well-being, and QoL before and after the intervention, providing evidence on the potential benefits of integrating non-pharmacological therapies into CP management.

### 2.2. Study Setting

The study was conducted among patients who participated in the CP workshop at San Juan de Dios del Aljarafe Hospital (Bormujos, Seville, Spain) between November 2021 and May 2024 (post-pandemic editions 9 to 21). Each workshop enrolled 15–20 patients who met the inclusion criteria: non-oncologic CP (musculoskeletal, visceral, post-traumatic, or neuropathic) persisting for more than 3 months and unresponsive to standard pharmacological treatment, surgery, or procedures performed by the reference Pain Unit.

### 2.3. Participants

**Figure 1:**
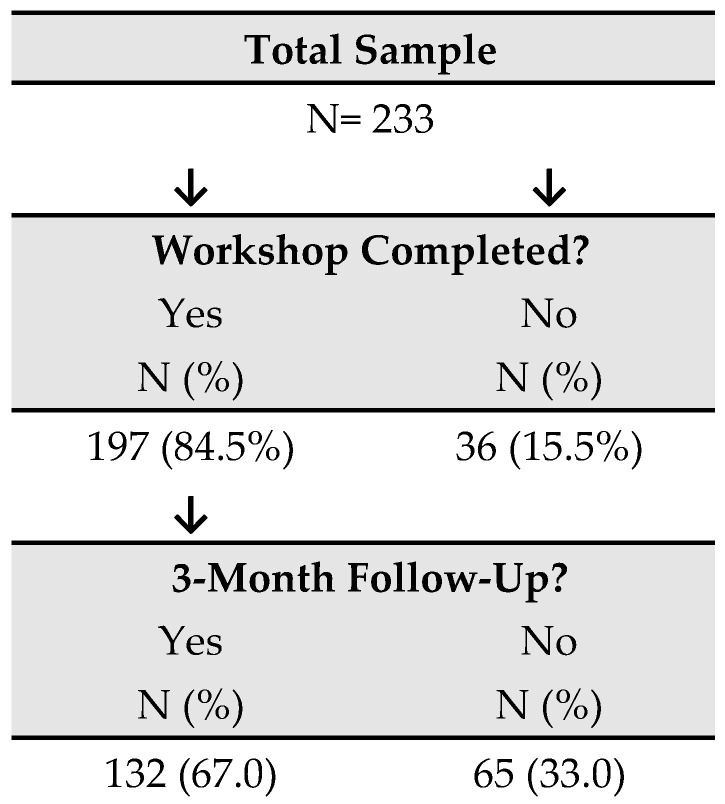
Participant flowchart.

A total of 233 patients participated in the workshops, of whom 197 completed the program (84.5%); the remaining 36 (15.5%) were excluded from the analysis. Among the 197 participants included in the study, 132 (67%) completed the 3-month follow-up assessments (Figure 1).

The reasons for withdrawal were as follows: loss of interest after two sessions (n = 2); the need to care for an ill family member (n = 2); acute illness of the patient (n = 1); inability to remain seated for prolonged periods (n = 1); changes in work schedule preventing attendance (n = 1); incomplete post-workshop documentation (n = 2); and discontinuation without explanation (n = 27). Participants did not attend any other multicomponent pain-management workshops before, during, or after the intervention period, including the 3-month follow-up. Each participant attended a single workshop, and each group consisted exclusively of newly enrolled patients. Once the workshop concluded, the intervention was considered complete; only a 3-month follow-up assessment was conducted to determine the persistence of effects.

### 2.4. Instruments

For data collection, participants completed a self-administered questionnaire at baseline that captured demographic information, pain characteristics, pharmacological treatment, and prior use of NPTs.

Various standardized scales were administered before the workshop (baseline), at the end of the intervention (1 month), and at 3-month follow-up. All standardized instruments used in this study therefore relied on validated Spanish versions with documented reliability and validity evidence:

- Pain intensity was measured using the *Numeric Pain Rating Scale* [24] (NPRS; 0 = no pain, 10 = worst imaginable pain), a measure widely used in CP research and clinical practice. The NPRS, which is language-independent and requires only numerical responses, has repeatedly shown high test–retest reliability and strong convergent validity with other pain intensity measures, as well as good sensitivity to clinically meaningful change in CP populations. The minimally clinically important difference (MCID) is ≥2 points.
- Subjective well-being was assessed with *Numeric Well-being Scale* [25] (*NWBS*; (0 = worst possible well-being, 10 = best possible well-being), a single-item global indicator developed for clinical use in our program that mirrors the structure of the NPRS to facilitate patient comprehension and minimize response burden. Although this item has not been formally validated as a stand-alone instrument in Spanish, its format is consistent with single-item ratings of QoL and related constructs, for which prior research has reported acceptable temporal stability and convergent validity with multi-item scales in population-based samples. As this is not a formally validated item, thresholds are extrapolated from the NPRS and other global numeric scales; the most reasonable and defensible criterion is an improvement of ≥1.5–2 points.
- Health-related quality of life (HRQoL) was evaluated with the *EuroQol-5D (EQ-5D)* [26], which includes five dimensions (mobility, self-care, usual activities, pain/discomfort, anxiety/depression) and provides both an index value (0–1, with higher scores indicating better health) and a visual analogue scale for self-perceived health status (0–100). The Spanish version of the EQ-5D has demonstrated good feasibility, internal consistency, and construct validity in primary care and population surveys (Intraclass correlation coefficient 0.70; Missing data 1.5%, ceiling effect 67% at 11,111, in the Catalan general population study). In CP populations, improvements of ≥0.05 of the index value are considered clinically relevant, and for the EQ-VAS (0–100), the accepted MCID is 7–10 points.
- Global self-esteem was assessed using the *Rosenberg Self-Esteem Scale* [27] (RSES), scored on a 4-point Likert scale and yielding total scores from 9 to 36 in this study. The Spanish adaptation of the RSES has shown a primarily unidimensional structure, high internal consistency (Cronbach’s α = 0.87–0.88), satisfactory test–retest reliability (r ≈ 0.84–0.85), and convergent validity with related self-concept dimensions in both community and clinical Spanish-speaking samples. There is no universal MCID for self-esteem, but longitudinal studies typically consider a difference of ≥2–3 points in the total score as a meaningful change.
- Resilience was measured with the *Brief Resilience Scale* [28] (BRS), which comprises six items rated on a 5-point Likert scale (total score range: 6–30), with higher scores indicating greater perceived ability to “bounce back” from stress. The Spanish version of the BRS has demonstrated good internal consistency (Cronbach’s α = 0.83), adequate test–retest reliability (0.69), and evidence of convergent, discriminant, and predictive validity in heterogeneous adult samples. This scale also lacks a formal MCID. In clinical reporting, a threshold of ≥1.5 points is commonly used to indicate a significant change.
- Anxiety and depressive symptoms were assessed with the *Hospital Anxiety and Depression Scale* [29] (HADS), a 14-item instrument with two 7-item subscales (anxiety and depression), each scored from 0 to 21. The Spanish adaptation of the HADS has shown a stable two-factor structure corresponding to its anxiety and depression subscales, satisfactory internal consistency (Cronbach’s alpha coefficients typically above 0.80), good test–retest reliability (0.896), and adequate criterion and construct validity for screening mood and anxiety disorders in Spanish medical and general population samples. The literature is quite consistent, placing the MCID between 1.5 and 1.7 points per subscale. More pragmatically, many trials use ≥2 points as the threshold for clinically relevant improvement.

Moreover, Patient-Reported Outcome Measures (PROMs) assessing pain management, medication use, habits, mood, and perceived usefulness of the techniques were included to capture subjective changes in domains central to patient-centered CP evaluation. These instruments were selected based on their widespread use in multicomponent CP interventions; their sensitivity to change across emotional, functional, and QoL domains; and their consistency with the multidimensional conceptualization of CP.

### 2.5. Procedure

The intervention consisted of a multicomponent, group-based psychoeducational and self-care training workshop focused on pain and emotion regulation. The approach emphasized active patient engagement in the management of their condition, with the aim of enhancing well-being and QoL (Table 1).

The workshop was delivered by a multidisciplinary team composed of three physicians (specialists in Physical Medicine and Rehabilitation, Internal Medicine, and Preventive Medicine), one psychologist, one physiotherapist, and one nurse, all professionals work at the San Juan de Dios del Aljarafe Hospital (Bormujos, Seville, Spain). All professionals had specific training in the non-pharmacological management of CP, enabling them to provide psychoeducation and evidence-based, low-risk therapeutic strategies. In addition, patients who had completed the workshop in previous editions participated as peer contributors, sharing their experiences with newly enrolled participants.

The workshop consisted of five consecutive weekly sessions, each lasting 3.5 h. The sessions were interactive and practical. Techniques such as relaxation, mental analgesia (meditation aimed at reducing pain), self-healing meditations, forgiveness, self-esteem, identification and reformulation of negative thoughts and limiting beliefs, motivation, creative visualization, and reinforcement of healthy habits (diet, exercise, sleep, stress management, cessation of toxic substances) were employed. Forgiveness of others and oneself, self-knowledge, and life purpose were promoted.

At the end of each session, patients were assigned weekly home-based tasks. Adherence and correct performance were reviewed at the beginning of the subsequent session, and any questions or difficulties were addressed.

The multicomponent workshop integrates evidence-based non-pharmacological techniques commonly employed in CP management. Relaxation exercises and guided self-healing meditations (e.g., mental analgesia, self-healing) correspond to mindfulness-based and mind–body interventions, which have demonstrated efficacy in reducing pain intensity and emotional distress [15,30]. Techniques aimed at enhancing self-esteem, identifying and reframing negative thoughts, and fostering forgiveness and self-forgiveness align with cognitive-behavioral strategies that improve coping, emotion regulation, and self-efficacy [31]. Acceptance, self-knowledge, and values-based work reflect principles of Acceptance and Commitment Therapy, which has demonstrated effectiveness in individuals with CP and emotional symptoms [14]. Compassion-focused elements, including practices of self- and other-directed forgiveness, are supported by evidence demonstrating beneficial effects of compassion-based approaches in CP [32]. Creative visualization and motivational strategies function as established psychological tools that facilitate adaptive coping and promote engagement in health-supporting behaviors [31]. Finally, lifestyle-oriented components, addressing diet, physical activity, sleep hygiene, stress management, and cessation of harmful substances, are consistent with current recommendations for comprehensive CP care and are routinely incorporated into multicomponent self-management programs [15,19].

Homework was assigned in each session. Patients daily recorded three key activities (mental analgesia, self-healing meditation, mirror affirmations), pain control, and analgesic needs. Patients from previous workshops were also invited to share their experiences.

At the end of the workshop, participants received a tool guide to facilitate continued practice of the techniques. A follow-up session was conducted one month later to reinforce learning and support adherence. At 3 months, outcome measures were reassessed, and two additional sessions focused on self-esteem and motivation to sustain behavioral change were delivered. Healthcare resource utilization, including emergency department visits, scheduled outpatient consultations, and medication use, was evaluated for the 4 months preceding and the 4 months following the intervention.

### 2.6. Data Analysis

Data were analyzed using SPSS v.27.0. Categorical variables were summarized as frequencies and percentages. Continuous variables were described as means and standard deviations (SD) or medians and interquartile ranges (IQR), according to distribution.

Between-group comparisons were conducted using Chi-square or Fisher’s exact tests for categorical variables and Student’s *t*-tests or Mann–Whitney U tests for continuous variables. Within-group pre–post comparisons were performed using paired *t*-tests or Wilcoxon tests. Statistical significance was defined as *p* < 0.05.

Effect sizes: For outcomes analysed with the paired Student’s *t*-test, the effect size Cohen’s d is reported and was calculated using the standard deviation of the paired differences (0.20 small, 0.50 medium, ≥0.80 large). Effect sizes are reported as absolute values; the direction of change (improvement or worsening) is indicated by the sign of the mean or median differences. For outcomes analysed with the Wilcoxon signed-rank test, the effect size r is reported and was calculated as r = Z/√N (Rosenthal), which can be interpreted similarly to a correlation coefficient (0.10 small, 0.30 medium, ≥0.50 large).

The project received approval from the Andalusian Biomedical Research Ethics Committee (code: 0213-N-22; 17 February 2022). Ethical guidelines of the Declaration of Helsinki and Organic Law 3/2018 on Personal Data Protection were followed. Written informed consent was obtained from all participants prior to inclusion in the study. Personal data were not disclosed to third parties. The workshop is registered as a scientific work in the Territorial Intellectual Property Registry (No. 04/2024/3397, 1 March 2024).

## 3. Results

The main outcomes at post-intervention and at the 3-month follow-up are presented below. Workshops conducted between November 2021 and May 2024 were evaluated; 197 patients completed the program, and 132 (67.0%) were assessed at 3 months.

Baseline characteristics of the 197 patients who completed the workshop were compared with those of the 36 who discontinued (Table 2). No statistically significant differences were observed between groups except for the following: participants who discontinued had a lower proportion of back pain (13.9% vs. 31.5%; *p* = 0.032); a higher proportion with no formal education (15.6% vs. 4.9%) or only primary education (37.5% vs. 23.5%) (*p* = 0.015); a lower proportion of individuals not working outside the home (5.9% vs. 22.0%); and a lower proportion reporting belief in the benefits of non-pharmacological therapies prior to the workshop (73.5% vs. 88.8%; *p* = 0.027). Regarding baseline measures, the only significant difference was higher initial pain intensity among those who did not complete the workshop (8.0 [6.0–9.0] vs. 7.0 [5.0–8.0]; *p* = 0.004) (Table 2).

At 3 months, 132 patients completed the follow-up assessments, while 65 did not. Comparison between these groups showed no statistically significant differences except for a higher proportion of individuals not working outside the home (36.7% vs. 15.2%; *p* = 0.002) and a slightly higher first quartile for baseline pain (7.0 [6.0–8.0] vs. 7.0 [5.0–8.0]; *p* = 0.037) (Table 2).

A group of 197 patients who completed the workshop participated in the study, 178 (90.4%) were women. The median age was 55 years. The educational level was mainly Primary (43; 21.8%); Secondary (25; 13.7%) or Baccalaureate/Vocational Training (67; 34.0%). 16 (8.8%) lived alone. 39 (21.6%) required a caregiver. The most frequent diseases were fibromyalgia (114; 57.9%), back pathology (62; 31.5%), and osteoarthritis (44; 22.3%). In total, 109 (55.3%) had generalized pain. Of the 197 who completed the workshop, 132 were followed up at three months. In workshops 9 to 17, the impact on healthcare resource consumption was evaluated (n = 107) (Table 3).

Pain decreased from 7.0 to 5.0 (median) at the end of the workshop (3 months: 5.0); well-being improved from 4.0 to 6.0 (3 months: 5.0); QoL increased from 0.399 to 0.581 (3 months: 0.556); health perception increased from 40.0 to 60.0 (3 months: 60.0); self-esteem increased from 23.5 to 27.1 (3 months: 26.6); resilience increased from 17.0 to 18.0 (3 months: 18.0); anxiety decreased from 13.0 to 9.0 (3 months: 11.0); and depression decreased from 11.4 to 7.2 (3 months: 6.8) (Table 3).

Effect sizes were large (r ≥ 0.50 or d ≥ 0.80) both at the end of the workshop and at 3 months for pain (r = 0.75 [95% CI: 0.67–0.81]; 3 months: r = 0.70 [0.59–0.79]), well-being (r = 0.65 [0.55–0.73]; 3 months: r = 0.56 [0.41–0.68]), perceived health status (r = 0.65 [0.55–0.73]; 3 months: r = 0.58 [0.43–0.69]), and anxiety (r = 0.62 [0.46–0.74]; 3 months: r = 0.59 [0.36–0.75]). QoL (d = 0.85 [0.68–1.01]; 3 months: d = 0.66 [0.47–0.85]) and self-esteem (d = 0.80 [0.63–0.97]; 3 months: d = 0.54 [0.35–0.73]) showed large effects post-intervention and medium effects at 3 months. Resilience showed a medium effect at post-intervention (r = 0.36 [0.14–0.54]) and a large effect at 3 months (r = 0.60 [0.35–0.77]).

In the short term, immediately after the workshop, all outcomes improved significantly relative to baseline, with most changes reaching the MCID; only two outcomes fell slightly below the threshold (pain and resilience). Pain (NPRS) decreased by 1.8 points (MCID ≥ 2); well-being increased by 2.0 points (MCID ≥ 1.5–2); EQ-5D index increased by 0.180 (MCID ≥ 0.05); EQ-VAS (My Health) increased by 20.0 points (MCID ≥ 7–10); self-esteem (RSES) increased by 3.7 (MCID ≥ 2–3); resilience (BRS) increased by 1.0 (MCID ≥ 1.5); anxiety (HADS) decreased by 3.0 (MCID ≥ 2); and depression (HADS) decreased by 4.2 (MCID ≥ 2).

In the medium term (3 months), all indicators remained improved relative to baseline, although the magnitude of change was smaller. Pain, well-being, and perceived health status were below their respective MCID thresholds, whereas QoL, self-esteem, resilience, anxiety, and depression continued to meet or exceed the MCID.

Up to 189 patients completed PROMs at the end of the workshop, and 110 repeated them at 3 months. A total of 145 (76.7%) achieved pain reduction with the learned techniques at the end of the workshop (3 months: 88; 80.7%). Medication decreased in 140 (80.5%) (3 months: 82; 78.1%). Habits improved in 157 (87.2%) (3 months: 92; 87.6%), mainly: physical exercise, diet, time for oneself, and more relaxation/meditation. Mood improved, increasing the number of patients with “jolly” mood to 57 (30.2%) (3 months: 32; 29.1%) and “normal” to 88 (46.6%) (3 months: 49; 44.5%) (Table 4).

The study evaluating the impact on resource consumption was conducted on patients from workshops 9 to 17 (n = 107), and, when comparing emergency visits and scheduled consultations 4 months before and 4 months after the workshop, a statistically significant decrease was found. In total, 40 (37.4%) patients reduced emergency visits and 12 (11.2%) increased them; 40 (37.4%) decreased scheduled consultations and 22 (20.6%) increased them. The number of medications decreased from 3.0 (2.0–4.0) to 2.0 (1.0–3.8), *p* < 0.001, 4–6 months after the workshop. There were 40 (50.0%) patients who reduced analgesics, 28 (35.0%) reduced antidepressants/anxiolytics/relaxants, and 14 (17.5%) reduced mixed medications (Table 5).

Overall satisfaction with the workshop was 9.7 (SD: 0.63) (scale 0–10); recommendation degree 9.9 (SD: 0.52); content clarity 9.7 (SD: 0.90); activity convenience 9.6 (SD: 0.75); usefulness for pain management 9.0 (SD: 1.63); and usefulness for disease management 9.2 (SD: 1.32).

## 4. Discussion

### 4.1. Short- and Medium-Term Effectiveness

In the short term, immediately after the workshop, all measured outcomes improved significantly relative to baseline, with changes reaching the MCID for all variables except pain and resilience, for which values approached the threshold.

These results are consistent with other studies applying NPTs: pain [21,22,23] and analgesic consumption decreased [13,14,22], and well-being [15,30] and QoL improved [14,17,21], as did self-perception of health status, self-esteem, resilience, anxiety, and depression [14,23].

The application of the main technique, mental analgesia, reduced pain in just over three-quarters of the patients, and this allowed four-fifths of the sample to reduce their medication (mainly analgesics, but also antidepressants, anxiolytics, and muscle relaxants) [12], primarily the frequency of the doses. The vast majority improved their habits, as occurred in other studies [33].

In the medium term (3 months), patients showed improvements across all measurement scales compared with baseline; however, these changes were more modest, and only QoL, self-esteem, resilience, anxiety, and depression reached the MCID. Björnsdóttir et al. reported positive results up to 6 months in pain reduction and QoL improvement [21]; in other studies, improvements were not maintained [34,35].

PROMs indicated that improvements were sustained at 3 months, with similar proportions across all evaluated domains (reduced pain through the techniques, decreased medication use, improved habits, and a shift toward cheerful or normal mood rather than discouragement or depression).

Overall satisfaction (both global and domain-specific) approached the maximum score. In open-ended comments, participants expressed gratitude toward the instructors and collaborators and reported substantial positive changes in their lives, including a reappraisal of their condition and pain, enhanced self-care and self-esteem, and renewed motivation to pursue personal goals.

### 4.2. Healthcare Resource Utilization

The CP barometer in Spain has shown the high frequency of primary care, hospital, and emergency visits in patients with CP [3]. The evaluated workshop is a valid option, as almost 40% of patients reduced emergency visits and scheduled consultations for their CP and associated symptoms (anxiety, sleep problems) in the 4 months after completion.

The present findings support the hypothesis that the workshop may contribute to reduced healthcare resource utilization and a potentially favorable economic profile; however, the data are insufficient to establish cost-effectiveness.

### 4.3. Comparison with Similar Experiences and Workshop Uniqueness

This workshop format with an uninterrupted 9-year trajectory is uncommon in Spain. It is crucial to highlight the experience in Catalonia, led by psychologists, whose fundamental therapy consists of psychological techniques. Their publications emphasize multicomponent programs for fibromyalgia (FIBROWALK, NAT-FM) based on pain neuroscience education, therapeutic exercise, cognitive-behavioral therapy, and mindfulness [15,30,31]. More recently, they have applied programs for chronic fatigue syndrome (FATIGUEWALK) [17] and for chronic low back pain with depressive symptoms (IMPACT Study), using acceptance and commitment therapy [14]. Another experience is that in Castilla y León, led by physiotherapists, which combines pain neuroscience education and therapeutic physical exercise for spinal CP, showing significant improvements [10,11].

Our workshop includes, in addition to the therapies mentioned, others such as: healthy eating, health coaching, self-esteem improvement, techniques for forgiving others and oneself, creative visualization, and reflection on life purpose.

Multicomponent workshops for CP management have shown benefits in controlling pain intensity and pain catastrophising, as well as improving mood and QoL.

More specifically, several components of the workshop (e.g., pain education, relaxation, meditations, cognitive reframing) parallel multicomponent interventions that integrate pain neuroscience education (PNE) and exercise. Pragmatic randomized controlled trials in Spanish primary care have shown that PNE combined with therapeutic exercise improves pain, disability, catastrophizing, kinesiophobia, and emotional distress in patients with chronic spinal pain [10,11]. In the latter, mediation analyses indicated that reductions in kinesiophobia and distress partly explained improvements in disability and analgesic use, supporting fear-avoidance and emotional distress as key mechanisms. Similarly, multicomponent programs for fibromyalgia that combine PNE, exercise therapy, cognitive-behavioral strategies, mindfulness, and/or nature exposure (NAT-FM and WALK) have demonstrated moderate-to-large effects on functional impact, pain, fatigue, mood, self-efficacy, catastrophizing, and kinesiophobia [15,16,30,31]. Related multicomponent and multidisciplinary programs in chronic back pain and low back pain have also reported benefits in mobility, pain behavior, and psychological variables in real-world and primary care settings [23,34,35,36].

On the other hand, the acceptance, values, and compassion-focused elements of the workshop are grounded in psychological approaches applied specifically to CP with comorbid emotional symptoms. Videoconference-based group Acceptance and Commitment Therapy and Behavioral Activation for Depression have shown superiority over treatment as usual in reducing pain interference, pain catastrophizing, and depressive symptoms in patients with chronic low back pain and clinically relevant depression [14]. Qualitative work from the same research program suggests that these interventions operate by fostering acceptance, values-based action, cognitive shifts, and improved emotional regulation in people living with persistent pain. Likewise, group-based psychological interventions in primary care (including relaxation, cognitive restructuring, behavioral activation, and coping-skills training) have produced improvements in mood, pain-related impact, and global impression of change in patients with chronic non-cancer pain [34]. Our workshop integrates closely related cognitive-behavioral, acceptance-based, and compassion-focused components (e.g., cognitive reframing of limiting beliefs, work on self-esteem, forgiveness and self-forgiveness, motivational strategies), with the aim of modifying catastrophizing, fear, self-criticism, and perceived lack of control.

Finally, the lifestyle-related components of the workshop (healthy diet, physical activity, sleep hygiene, stress management, and reducing harmful substances) align with multicomponent self-management and rehabilitation programs that emphasize behavioral activation and health promotion in CP and chronic fatigue conditions [17,23,35]. Across these trials, improvements in physical activity, self-efficacy, and other behavioral markers have been associated with better pain-related outcomes and QoL.

### 4.4. Applicability and Practical Limitations

The workshop is highly adaptable to both public and private healthcare systems, at hospital and primary care levels. It requires minimal material resources but does require specific training for the professional team, which has already been provided to 30 primary care clinicians. The project has received research funding and institutional recognition.

A practical limitation was patient dropout, largely attributable to serious events (death or worsening of a family member) or severe exacerbation of the patient’s condition; in such cases, participants were offered the option to discontinue and join a later edition. Other withdrawals were related to scheduling incompatibilities with work or, in a minority of cases, distrust of the intervention; several participants discontinued without providing a reason.

A subgroup of patients was identified who were unwilling to engage actively in their recovery or who were primarily focused on obtaining sick leave or disability benefits. Incorporation of a motivational interviewing component could assist in identifying individuals unlikely to participate effectively and in reallocating their place to more engaged candidates.

Among participants who discontinued the workshop, only a few distinguishing characteristics were identified. Individuals with lower educational attainment, those not working outside the home, those reporting higher baseline pain, and those who did not believe in the benefits of non-pharmacological therapies appeared less likely to perceive the workshop as useful or had greater difficulty maintaining attendance. The differentiating findings among participants who did not complete the 3-month follow-up—slightly higher baseline pain and a higher proportion not working outside the home—were insufficient to support hypotheses regarding the reasons for loss to follow-up.

Most attrition occurred at the 3-month assessment, particularly in the earliest editions. To minimize these losses, workshops were subsequently scheduled to avoid summer months and periods with multiple holidays, and follow-up dates were agreed upon with each patient group.

Among those who did not succeed in reducing pain, the main reasons were inability to relax, high pain intensity, and the existence of a recent unresolved emotional grief. Nonetheless, overall participant satisfaction is near maximal, as those who did not manage to reduce pain still improved other aspects.

The sample was predominantly taken from women, and the types of CP and underlying conditions represented were highly heterogeneous. Fibromyalgia, spinal pathology, and osteoarthritis were the most prevalent conditions, frequently resulting in generalized or multi-site pain [12]. This diversity may be considered beneficial for participants, as it allows the sharing of experiences across different conditions that share a common symptom—CP—often accompanied by emotional distress. Nevertheless, many multicomponent CP programs are typically designed for patients with a single, specific pathology. [10,14,16,17].

However, given the characteristics of the participants included, the results cannot be generalized to other types of non-oncologic pain that were less represented in the sample, such as neuropathic or post-traumatic pain. A larger proportion of male participants would also be required to examine potential sex-related differences. According to a 2007 national survey, nearly four out of ten U.S. adults used non-pharmacological therapies in addition to conventional treatments for pain [37], and subsequent studies have shown that their use continues to increase, particularly among women and individuals with higher educational attainment [38,39].

## 5. Conclusions

The evaluated multicomponent workshop, a non-pharmacological intervention for the management of non-oncologic CP, generated encouraging, hypothesis-generating findings within a real-world implementation context. The workshop format was associated with significant and sustained medium-term improvements in pain, mental health indicators (depression and resilience), and medication consumption, and demonstrated applicability across a heterogeneous patient population. Of particular relevance to the healthcare system, reductions of nearly 40% were observed in the use of outpatient consultations and emergency services for pain or related symptoms. These findings suggest potential benefits and underscore the need for more rigorous controlled evaluations, including study designs incorporating comparator groups.

## 6. Patents

The workshop is registered as a scientific work in the Territorial Intellectual Property Registry (No. 04/2024/3397, 1 March 2024).

## Figures and Tables

**Table 1:** Workshop Content and Facilitators.

**Session 1:**
1. Welcome. (Physician 1)
2. Assessment of workshop expectations (brainstorming). (Physician 1)
3. Physiopathological mechanism of pain. Differences between acute and chronic pain. (Physician 2)
4. Influence of emotions on pain intensity. (Physician 1)
5. Activities: My limiting beliefs; Mirror affirmations to motivate change; Mental analgesia technique. (Physician 1)
6. Homework assignments. (Physician 1)
**Session 2:**
1. Sharing results of homework assignments. (Physician 1)
2. Activity: Repetition of the mental analgesia technique. (Physician 1)
3. Pain and its impact on the patient’s life: “You are not your pain.” (Psychologist)
4. Forgiveness and self-forgiveness. Ho’oponopono technique. Some experiences. (Physician 1)
5. Self-esteem. What is it? Tools that increase self-confidence. (Physician 1)
6. Activities: Limiting labels; Self-esteem mirror affirmation; Reading “When I Truly Loved Myself”; Self-healing meditation; Examples of individuals with self-improvement experiences (video and real patient testimony). (Physician 1)
7. Motivation for change. Life purpose. (Physician 1)
8. Homework assignments. (Physician 1)
**Session 3:**
1. Sharing results of homework assignments. (Physician 1)
2. Advice on healthy aging. (Physician 1)
3. Promotion of healthy habits: General approach (Physician 1); Healthy eating (Nutritionist and Nurse); Physical exercise (Physiotherapist); Sleep quality (Nurse).
4. Activities: Self-assessment of habits; Metta Meditation; Connecting with one’s being; Motivation for change (“Awakening the Senses”). (Physician 1)
5. Experience shared by a patient from a previous workshop. (Patient Contributor)
6. Homework assignments. (Physician 1)
**Session 4:**
1. Sharing results of homework assignments. (Physician 1)
2. Active participation in my own healing. How to cope with illness. (Physician 3)
3. Tool: Creative visualization. (Physician 1)
4. How to improve self-esteem. (Physician 1)
5. Experience shared by a patient from a previous workshop. (Patient Contributor)
6. Activities: Self-esteem meditation; Self-connection through music: “El eterno sol.” (Physician 1)
7. Homework assignments. (Physician 1)
**Session 5:**
1. Sharing results of homework assignments. (Physician 1)
2. Tool: Examples of individuals with self-improvement experiences (videos of people who are role models for personal growth). (Physician 1)
3. Experience shared by a patient from a previous workshop. (Patient Contributor)
4. Review of tools explained during the workshop. Sharing. Question and answer session. (Physician 1)
5. Workshop evaluation and completion of scales. (Physician 1)
6. Delivery of the Guide with tools presented in the workshop and other advice. (Physician 1)
7. Activities: Gratitude to patients for their trust in the workshop and invitation to continue applying and deepening the tools to control pain and better cope with the illness; Final farewell. (Physician 1).

**Table 2:** Sample Characteristics.

		Workshop Completed			3-Month Follow-Up		
Variables		Yes	No	Signifi-cance (p)	Yes	No	Signifi-cance (p)
		N (%)	N (%)		N (%)	N (%)	
Total Participants		197 (84.5)	36 (15.5)		132 (67.0)	65 (33.0)	
Sex	Woman (yes)	178 (90.4)	30 (83.3)	0.239 ^a^	122 (92.4)	56 (86.2)	0.161 ^b^
Pain-causing condition	Fibromyalgia (yes)	114 (57.9)	23 (63.9)	0.500 ^b^	82 (62.1)	32 (49.2)	0.085 ^b^
	Osteoarthritis (yes)	44 (22.3)	10 (27.8)	0.477 ^b^	30 (22.7)	14 (21.5)	0.851 ^b^
	Spine disorder (yes)	62 (31.5)	5 (13.9)	0.032 ^b^*	39 (29.5)	23 (35.4)	0.407 ^b^
	Migraine (yes)	13 (6.6)	3 (8.3)	0.720 ^a^	8 (6.1)	5 (7.7)	0.762 ^a^
	Autoimmune diseases (yes)	11 (5.6)	0 (0)	0.222 ^a^	8 (6.1)	3 (4.6)	1 ^a^
	Joint damage (yes)	29 (14.7)	3 (8.3)	0.432 ^a^	17 (12.9)	12 (18.5)	0.298 ^b^
	Neuropathic injury (yes)	14 (7.1)	1 (2.8)	0.478 ^a^	9 (6.8)	5 (7.7)	0.777 ^a^
	Chronic fatigue (yes)	23 (11.7)	1 (2.8)	0.139 ^a^	19 (14.4)	4 (6.2)	0.090 ^b^
	Widespread pain (yes)	166 (84.3)	31 (86.1)	0.778 ^b^	115 (87.1)	51 (78.5)	0.117 ^b^
	Other (yes)	35 (17.8)	5 (13.9)	0.571 ^b^	23 (17.4)	12 (18.5)	0.858 ^b^
Educational level	No formal education	9 (4.9)	5 (15.6)		5 (4.0)	4 (7.0)	
	Primary education	43 (23.5)	12 (37.5)		30 (23.8)	13 (22.8)	
	Secondary education	25 (13.7)	5 (15.6)		17 (13.5)	8 (14.0)	
	High school/Intermediate vocational training	67 (36.6)	9 (28.1)	0.015 ^b^*	50 (39.7)	17 (29.8)	0.595 ^b^
	University degree (Diploma/Bachelor’s/Graduate)	39 (21.3)	1 (3.1)		24 (19.0)	15 (26.3)	
Employment status	Full-time employment	25 (13.7)	8 (25.0)		19 (15.1)	6 (10.5)	
	Part-time employment	4 (2.2)	3 (9.4)		3 (2.4)	1 (1.8)	
	Employed, on short-term sick leave	7 (3.8)	1 (3.1)		3 (2.4)	4 (7.0)	
	Employed, on long-term sick leave	55 (30.1)	4 (12.5)	0.069 ^b^	41 (32.5)	14 (24.6)	0.511 ^b^
	Retired/pensioner	3 (1.6)	0 (0)		2 (1.6)	1 (1.8)	
	Not working outside the home	89 (48.6)	16 (50.0)		58 (46.0)	31 (54.4)	
Need for a caregiver	No	141 (78.3)	26 (83.9)		96 (77.4)	45 (80.4)	
	Yes, part-time	33 (18.3)	5 (16.1)	0.547 ^b^	23 (18.5)	10 (17.9)	0.727 ^b^
	Yes, full-time	6 (3.3)	0 (0)		5 (4.0)	1 (1.8)	
Caregiver of another person	No	142 (79.3)	25 (78.1)		99 (79.8)	43 (78.2)	
	Yes, part-time	20 (11.2)	2 (6.3)	0.446 ^b^	15 (12.1)	5 (9.1)	0.551 ^b^
	Yes, full-time	17 (9.5)	5 (15.6)		10 (8.1)	7 (12.7)	
Work absenteeism	No	34 (18.4)	8 (25.0)		26 (21.0)	8 (13.1)	
	Yes, sometimes	52 (28.1)	9 (28.1)		36 (29.0)	16 (26.2)	
	Yes, often	51 (27.6)	10 (31.3)	0.585 ^b^	34 (27.4)	17 (27.9)	0.372 ^b^
	Not working outside the home	48 (25.9)	5 (15.6)		28 (22.6)	20 (32.8)	
Cause of sick leave	No	36 (19.5)	11 (34.4)		30 (24.0)	6 (10.0)	
	Yes	108 (58.4)	19 (59.4)	0.007 *^b^	76 (60.8)	32 (53.3)	0.002 *^b^
	Not working outside the home	41 (22.2)	2 (6.3)		19 (15.2)	22 (36.7)	
	*No response*	*12 (6.1)*	*4 (11.1)*		*7 (5.3)*	*5 (7.7)*	
Baseline mood	Happy	6 (3.1)	1 (2.8)		6 (4.6)	0 (0)	
	Neutral	48 (24.6)	7 (19.4)		30 (22.9)	18 (28.1)	
	Discouraged	72 (36.9)	13 (36.1)	0.887 ^b^	52 (39.7)	20 (31.3)	0.173 ^b^
	Depressed	69 (35.4)	15 (41.7)		43 (32.8)	26 (40.6)	
Use of analgesics	Yes	163 (88.1)	25 (96.2)	0.322 ^a^	118 (89.4)	45 (84.9)	0.394 ^b^
	*No response*	*12 (27.8)*	*10 (6.1)*		*0 (0)*	*12 (18.5)*	
Use of antidepressants, anxiolytics, or sedatives	Yes	133 (72.3)	15 (57.7)		95 (72.5)	38 (71.7)	
	*No response*	*12 (27.8)*	*10 (6.1)*	0.127 ^b^	*0 (0)*	*12 (18.5)*	0.910 ^b^
Has ever used morphine	Yes	102 (54.3)	20 (58.8)	0.622 ^b^	64 (51.2)	38 (60.3)	0.236 ^b^
Belief in the benefits of non-pharmacological therapies	Yes	166 (88.8)	25 (73.5)	0.027 ^a^*	110 (86.6)	56 (93.3)	0.174 ^a^*
Age (Mean (SD^e^))		54.7 (11.45)	52.9 (12.22)	0.392 ^c^	54.0 (11.70)	55.92 (10.90)	0.278 ^c^
Baseline pain (Median (Q1–Q3)^f^)	Scale 0–10	7.0 (5.0–8.0)	8.0 (6.0–9.0)	0.004 ^d^*	7.0 (5.0–8.0)	7.0 (6.0–8.0)	0.037 ^d^*
Baseline well-being (Median (Q1–Q3)^f^)	Scale 0–10	4.0 (2.0–4.5)	3.0 (2.0–4.4)	0.081 ^d^	4.0 (2.0–4.0)	4.0 (3.0–5.5)	0.245 ^d^
Baseline health status	Scale 0–100	40.0 (30.0–60.0)	40.0 (30.0–50.0)	0.553 ^d^	45.0 (30.0–60.0)	40.0 (28.8–50.0)	0.101 ^d^
Baseline quality of life (EQ-5D) (Mean (SD^e^))	Scale 0–1	0.400 (0.254)	0.349 (0.230)	0.264 ^c^	0.409 (0.246)	0.378 (0.269)	0.428 ^c^
Baseline self-esteem (Mean (SD^e^))	Scale 9–36	23.5 (5.22)	23.4 (6.30)	0.896 ^c^	23.6 (5.18)	23.3 (5.36)	0.690 ^c^
Baseline resilience ** (Median (Q1–Q3)^f^)	Scale 6–30	17.0 (14.0–19.0)	18.0 (14.8–19.3)	0.520 ^d^	17.0 (13.0–18.0)	18.0 (16.5–19.0)	0.077 ^d^
Baseline anxiety ** (Median (Q1–Q3)^f^)	Scale 0–21	13.0 (11.0–16.0)	12.0 (8.8–15.5)	0.658 ^d^	13.0 (11.0–16.0)	12.0 (10.0–17.0)	0.928 ^d^
Baseline depression** (Mean (SD^e^))	Scale 0–21	11.4 (4.03)	10.7 (5.19)	0.511 ^c^	11.1 (4.10)	12.0 (3.91)	0.302 ^c^

* Statistical significance *p* < 0.05. ** Smaller sample size, n = 84 (this questionnaire was not administered in the earliest editions). ^a^ Fisher’s exact test; ^b^ Chi-square test; ^c^ Independent-samples Student’s *t*-test; ^d^ Mann–Whitney U test for independent samples; ^e^ SD: Standard Deviation; ^f^ Q1: Quartile 1; Q3: Quartile 3.

**Table 3:** Main Results. Measurement Scales.

	Health Indicators (Scale)	Baseline	N	Post-Workshop	N	*p*	At 3 Months	N	*p*
Pain (0–10)	Median (IQR)	7.0 (5.0–8.0)	197	5.0 (4.0–6.0)	194	<0.001 *^a^	5.0 (4.0–6.0)	130	<0.001 *^a^
Differences	Median (IQR)/Effect size (95% CI) ^c^			−1.8 (−3.0–0)	*r = 0.75 (0.67–0.81)*		−1.0 (-3.0–0)	*r = 0.70 (0.59–0.79)*	
Well-being (0–10)	Median (IQR)	4.0 (2.0–4.5)	197	6.0 (4.0–7.0)	194	<0.001 *^a^	5.0 (4.0–6.0)	129	<0.001 *^a^
Differences	Median (IQR)/Effect size (95% CI) ^c^			2.0 (0–3.0)	*r = 0.65 (0.55–0.73)*		1.0 (0–3.0)	*r = 0.56 (0.41–0.68)*	
My health (0–100)	Median (IQR)	40.0 (30.0–60.0)	193	60.0 (50.0–75.0)	193	<0.001 *^a^	60.0 (45.0–79.0)	127	<0.001 *^a^
Differences	Median (IQR)/Effect size (95% CI) ^c^			20.0 (0–30.0)	*r = 0.65 (0.55–0.73)*		10.0 (0–30.0)	*r = 0.58 (0.43–0.69)*	
Quality of life (0–1)	Mean (SD)	0.399 (0.2535)	194	0.581 (0.2406)	193	<0.001 *^b^	0.556 (0.2584)	129	<0.001 *^b^
Differences	Median (IQR)/Effect size (95% CI) ^d^			0.180 (0.2116)	*d = 0.85 (0.68–1.01)*		0.145 (0.219)	*d = 0.66 (0.47–0.85)*	
Self-esteem (9–36)	Mean (SD)	23.5 (5.22)	182	27.1 (4.76)	179	<0.001 *^b^	26.6 (5.20)	125	<0.001 *^b^
Differences	Median (IQR)/Effect size (95% CI) ^d^			3.7 (4.67)	*d = 0.80 (0.63–0.97)*		2.7 (5.00)	*d = 0.54 (0.35–0.73)*	
Resilience (6–30)	Median (IQR)	17.0 (14.0–19.0)	84	18.0 (16.0–20.0)	83 ^e^	0.002 *^a^	18.0 (17.0–19.0)	49 ^e^	<0.001 *^a^
Differences	Median (IQR)/Effect size (95% CI) ^c^			1.0 (-1.0–4.3)	*r = 0.36 (0.14–0.54)*		2.0 (0–5.5)	*r = 0.60 (0.35–0.77)*	
Anxiety (0–21)	Median (IQR)	13.0 (11.0–16.0)	84	9.0 (7.0–14.0)	83 ^e^	<0.001 *^a^	11.0 (7.0–12.5)	49 ^e^	<0.001 *^a^
Differences	Median (IQR)/Effect size (95% CI) ^c^			−3.0 (−5.0–0)	*r = 0.62 (0.46–0.74)*		−3.0 (-5.5–0)	*r = 0.59 (0.36–0.75)*	
Depression (0–21)	Mean (SD)	11.4 (4.03)	84	7.2 (4.27)	83 ^e^	<0.001 *^b^	6.8 (4.36)	49 ^e^	<0.001 *^b^
Differences	Median (IQR)/Effect size (95% CI) ^d^			−4.2 (3.98)	*d = 1.06 (0.79–1.33)*		−3.8 (4.80)	*d = 0.79 (0.46–1.11)*	

IQR = Interquartile Range. SD = Standard Deviation. * Statistical significance *p* < 0.05. ^a^ Wilcoxon test; ^b^ Paired Student’s *t*-test; ^c^ and ^d^ Effect size Post-Workshop and at 3 months vs. Baseline (95% CI); ^c^ For outcomes analysed with the Wilcoxon signed-rank test, the effect size was calculated as r Rosenthal (0.10 small, 0.30 medium, ≥0.50 large); ^d^ For outcomes analysed with the paired Student’s *t*-test, the effect size Cohen’s d (0.20 small, 0.50 medium, ≥0.80 large) is reported; ^e^ These scales were introduced later, so first editions were not completed. Clarification: *p*-values reflect statistically significant differences between the means and medians of the two measured time points (post-workshop and at 3 months) compared to baseline values.

**Table 4:** Self-Assessment of Workshop Impact–Patient-Reported Outcomes (PROMs).

Questions/Indicators	Post-Workshop N (%)	3-Month Follow-Up N (%)
Pain decreased with technique application (N post-workshop = 189; N 3 months = 109)	145 (76.7)	88 (80.7)
Medication decreased after the workshop (N post-workshop = 174; N 3 months = 105)	140 (80.5)	82 (78.1)
Dosage frequency decreased	86 (61.4)	58 (70.3)
Doses decreased	67 (47.9)	38 (46.3)
Switched to a less potent medication	41 (29.3)	31 (37.8)
Stopped taking some medications	59 (42.1)	48 (58.5)
*Were not taking medication at baseline*	10	3
Improved Habits (N post-workshop = 180; N 3 months = 105)	157 (87.2)	92 (87.6)
Mood state at the end of the workshop (N post-workshop = 189; N 3 months = 110)		
Cheerful	57 (30.2)	32 (29.1)
Normal	88 (46.6)	49 (44.5)
Discouraged	31 (16.4)	22 (20.0)
Depressed	13 (6.9)	7 (6.4)
Total maximum sample size	189	110

**Table 5:** Impact on Resource Consumption.

**Resource**	**4 Months Before** **Median (IQR ^a^)**	**4 Months After** **Median (IQR ^a^)**	**Significance (*p*)**	**Observed Changes**
Emergency Department Consultations	0 (0–1.0)	0 (0–0)	<0.001 *	Decreased 40 (37.4%) No change 55 (51.4%) Increased 12 (11.2%)
Scheduled consultations	1.0 (0–2.0)	0 (0–1.0)	0.017 *	Decreased 40 (37.4%) No change 45 (42.1%) Increased 22 (20.6%)
**Resource**	**Workshop Baseline** Median (IQR ^a^)	**4 Months Post-Workshop** Median (IQR ^a^)	**Significance (*p*)**	**Observed Changes**
Total amount of medications	3.0 (2.0–4.0)	2.0 (1.0–3.8)	<0.001 *	Decreased 44 (55.0%) No change 21 (26.3%) Increased 15 (18.8%)
Analgesics	2.0 (1.0–3.0)	1.0 (0–2.0)	<0.001 *	Decreased 40 (50.0%) No change 28 (35.0%) Increased 12 (15.0%)
Antidepressants, Anxiolytics, Relaxants	1.0 (0–2.0)	1.0 (0–2.0)	0.025 *	Decreased 28 (35.0%) No change 37 (46.3%) Increased 15 (18.8%)
Mixed medications ^b^	0 (0–1.0)	0 (0–0)	0.023 *	Decreased 14 (17.5%) No change 63 (78.8%) Increased 3 (3.75%)

^a^ IQR = Interquartile Range; ^b^ Mixed medications: medications with analgesic and anxiolytic/antidepressant effects.* Statistical significance *p* < 0.05; Wilcoxon test applied.

## Data Availability

The original contributions presented in this study are included in the article. Further inquiries can be directed to the corresponding authors.

## Abbreviations

The following abbreviations are used in this manuscript:

CP	Chronic Pain
IQR	Interquartile Range
NPTs	Non-Pharmacological Therapies
PROM	Patient-Reported Outcome Measure
SD	Standard Deviation
QoL	Quality of Life
